# Extensive cosmic showers detection: the importance of timing and the role of GPS in the EEE experiment

**DOI:** 10.1007/s10291-021-01152-9

**Published:** 2021-07-10

**Authors:** Giancarlo Cerretto, Davide Calonico, Elena Cantoni, Filippo Levi, Alberto Mura, Marco Sellone

**Affiliations:** Time and Frequency Group, Quantum Metrology and Nanotechnologies Division (QN), Istituto Nazionale Di Ricerca Metrologica (I.N.RI.M.), Strada delle Cacce 91, 10135 Torino, Italy

**Keywords:** Astrophysics, Cosmic rays, Extensive air showers, EEE, Time, UTC

## Abstract

Extreme Energy Events (EEE) is an extended Cosmic Rays (CRs) Observatory, composed of about 60 tracking telescopes spread over more than 10 degrees in Latitude and Longitude. We present the metrological characterization of a representative set of actually installed EEE GPS receivers, their calibration and their comparison with respect to dual-frequency receivers for timing applications, as well as plans for a transportable measurement system to calibrate the currently deployed GPS receivers. Finally, the realization of an INRIM Laboratory dedicated to EEE, aimed at hosting reference telescopes and allowing timing studies for Particle Physics/Astrophysics experiments, is presented, as well as the possibility of synchronizing already deployed telescopes utilizing White Rabbit Technique, over optical fiber links, directly with the Universal Time Coordinated time scale, as realized by INRIM (UTC(IT)).

## Introduction

CRs are high-energy nuclei coming from outer space and interacting with the earth’s atmosphere. Their energy spectrum spans ten orders of magnitude from 10^10^ to 10^20^ eV, the peculiarities of its different regions having important astrophysical implications. When we talk about High-Energy Cosmic Rays (HECRs), the region of the spectrum explored is 10^18^ eV and more. Here, the transition from galactic to the extragalactic origin of CRs is supposed to take place and the study of the CRs characteristics can help to discriminate between different possible astrophysical scenarios related to their origin and propagation (Allard 2007). At higher energies, the ambition of the researchers is to find a way to reconstruct the arrival direction of a few particles correlated with some specific source. This is typically done through anisotropy studies, where possible statistical variances from the basically isotropic flux are investigated in different space vault regions (The Pierre Auger Collaboration 2015).

CRs are called primary particles because when they hit the top of the atmosphere, hadronic collisions occur with air nuclei, giving way to the formation of the Extensive Air Showers (EAS), composed by thousands or even millions of secondaries. The particle avalanche in the EAS has some main components, hadronic, electromagnetic, fluorescence effects, and among these, the muon component (leptons, positive and negative charge, mass about 106 MeV/c^2^), being the most penetrating one, is able to reach the sea-level. At the considered energies, EAS are typically measured exploiting large arrays of detectors at the ground, aiming to infer indirectly the characteristics of the primaries—typically arrival direction, energy, and mass—from those of the EAS, mainly arrival direction and the number of particles. Several experiments have deeply investigated the energy spectrum and the primary composition of high-energy cosmic radiation up to the Greisen–Kuzmin–Zatsepin (GKZ) cutoff (Gerasimova 1960). Among these, it is important to recall the Pierre Auger experiment (The Pierre Auger Collaboration 2015).

One of the most intriguing scientific quest related to the Ultra-High-Energy Cosmic Rays (UHECR), is the search for EAS correlations at large distances (LDC). The existence of such a physical phenomenon has been proposed in a set of theoretical scenarios, i.e., heavy nuclei photon-induced fragmentation in the neighborhoods of stars or particle pairs production at the source. This search requires muon detectors, displaced at distances larger than the typical EAS footprint at the ground (a few km), to be able to measure the absolute time with high accuracy. The very low expected LDC rate (few events per year over about 10^5^ km^2^), is the main limit to the LDC search, where the time accuracy plays a fundamental role in background reduction.

In such a frame, the present work is based, dealing with the EEE experiment, an international experiment whose main purpose is detecting EAS, going into the detail of the detectors used and the importance of timing for this type of measurements. The preliminary activities aimed at improving the time system of the experiment and carried out by INRIM are presented, together with the perspectives of a three-year formal collaboration recently established between INRIM and the EEE Collaboration.

## EEE experiment

The EEE Observatory (Abbrescia 2013) is an extended CRs array involving several Research Institutes: the Centro Ricerche Enrico Fermi (CREF), the INFN (Italian National Institute for Nuclear Physics) and MIUR (Italian Ministry of Education, University and Research). The extension of the array and the technology of the muon detectors allow for addressing several CRs-related scientific investigations, besides the search for LDCs: HECR, solar physics, climate investigations, etc. The observatory also plays a fundamental role in Science Dissemination, being the 60 detectors installed in high schools and Laboratories (i.e., CERN, INFN, CREF) over more than 3 × 10^5^ km^2^.

The experimental approach for the investigations on LDCs is the search for correlations, in terms of times of arrival and EAS cores alignment, between different EASs at large distances. This search depends as well on the array capability of reconstructing single muons (and subsequently local EAS) from individual muon tracks, therefore measuring their arrival time and direction with high accuracy.

At present, the sparse distribution of the EEE detectors makes the observatory a unique facility in order to investigate the existence of LDCs phenomena, with a surface area of about two orders of magnitude greater than that of the largest other arrays. The EEE collaboration already showed weak hints of LDCs, out of a dataset covering one year of exposure, at present at a low statistical confidence level (Abbrescia 2017, 2018). The use of the whole exposure dataset (few years) and an improved timing performance could open to a sizeable increase in the discovery potential.

## EEE telescopes and events time tagging

The EEE telescopes are based on the Multigap Resistive Plate Chambers (MRPC) technology, extensively used and developed for the ALICE experiment Time OF Flight (TOF) subsystem at the Large Hadron Collider (LHC). Three MRPC detector planes per telescope (Abbrescia 2016), each 1.3 m^2^, flushed with a mixture of C_2_H_2_F_4_ (98%) and SF_6_ (2%) and powered at 20 kV, are the main components of an EEE muon station. An ionizing particle (mainly muons) triggers an electron-avalanche while traveling through the gas gaps. The coordinates and the time measurements are allowed by a set of readout strips, 24 per MRPC plane (160 cm long, 3.2 cm pitch), together with left–right Front-End Acquisition (FEA) cards. Each avalanche induces two signals on the copper strips, readout by the FEA cards and sent to Time to Digital Converters (TDCs, 100 ps resolution, 25 ps max resolution), allowing for the event data acquisition. Subsequently, the muon hit displacement in space on each MRPC plane is derived by the left–right arrival time difference of the avalanche signals, the strip number, and the MRPC plane. Conversely, the events time tagging is measured by subdividing the 1PPS signal of a commercial single-frequency GPS receiver (presently not calibrated). The GPS 1PPS acts resetting the TDCs, while the TDCs provide the sub-second time tag. The three chambers coincidence gives the overall event trigger. Finally, the GPS NMEA string is used to time tag the triggering event, together with the TDC partial measurements. A new trigger system for EEE telescopes has been recently proposed, aimed at further improving the EEE detection capabilities (Panetta 2019).

## Importance of timing in EEE

As pointed out, the study of CR is generally demanding the search of coincidences. This search moves across a wide range of scales, both in terms of time and space. The search for EAS requires the identifications of muon tracks, their direction and arrival time: the coincidence within a few km and few microseconds between different muon tracks is an EAS candidate. The search for LDCs candidates (rare events, coincidences between different EAS at about 100–1000 km), is mainly limited by the coincidences between uncorrelated EAS. The so-called Spurious Coincidence Rate (RSC), that can be applied to both the search for correlated muon tracks, while searching for EAS, as well as to correlated EAS, while searching for LDCs. It can be expressed as:

$${\text{R}}_{{{\text{SC}}}} {{~\sim N \cdot }}\left( {{\text{R}}_{{{\text{SST}}}} } \right)^{{\text{N}}} {{~\cdot }}\left( {{{\Delta T}}} \right)^{{{\text{N - 1}}}}$$

where R_SC_ is Spurious Coincidence Rate that all telescopes will experience an event, N is the Number of Telescopes R_SST_ is the Single Telescope Rate of spurious events, and ΔT is the Time Coincidence Window, the maximum accepted time interval to consider muon tracks synchronous.

After imposing quality cuts on the reconstruction, the actual rate of good muon tracks is still several tens of Hertz: an average value of RSST = 20–40 Hz can be taken for single muons detected in each telescope (Abbrescia 2018). Accurate timing is then a key parameter for the suppression of spurious coincidences. As an example, the equation above shows how the spurious coincidence rate for a set of 4 telescopes is suppressed by a factor 8 if the accuracy ΔT improves by a factor 2. Furthermore, since the EAS arrival directions can be identified by the arrival time difference between muon tracks, a very accurate/precise time (i.e., nanosecond level), would allow a better accuracy in the arrival direction reconstruction. This would apply to both the EAS arrival direction and, as a consequence, to the LDCs candidates that may arise from far EAS coincidences or far multi-telescopes coincidences.

Therefore, an improved timing accuracy directly brings to an improved identification confidence level for LDCs candidates (Abbrescia 2018). As an example, a preliminary study for searching LDCs identified 40 candidates on an expected background of 23 events, therefore an excess of 17 events (La Rocca 2019). This result was obtained by searching in a dataset of 3 × 10^7^ coincident events measured by 42 telescopes in 5 clusters, taking into account telescopes at distances above 5 km with at least 3 muon tracks per event. The excess of 17 events was observed by reducing the time window from 1 s to 1 microsecond: a clear example of how timing plays a central role in searching rare events. This promising result is not yet a real hint of LDC existence, being the signal to noise ratio not yet enough to define a discovery. An additional improvement in timing would allow making a profit of the interplay between timing and tracking, thus reducing the maximum allowed coincidence window, enhancing the signal to noise, and finally, the discovery potential of the experiment.

## INRIM role in EEE

Starting from the concrete assumption that timing in EEE is fundamental, at the end of 2019 INRIM and CREF launched a collaboration aimed at improving the EEE timing system and disseminating knowledge about timing for the EEE collaboration. This collaboration resulted in a formal agreement signed by both Institutes. More in details, outreach has been provided through the INRIM participation to the “Tenth Centro Fermi Projects Conference”, with masterclasses about Metrology, Time Metrology, and on the use of GNSS for time synchronization, with contributions included in the International Physics Outreach Group database (The IPPOG official website: https://ippog.org/).

In parallel, the scientific role for INRIM relies on important activities spread over the three years of collaboration. Following an incremental approach, INRIM foresees to support the EEE collaboration with the metrological characterization of the telescope network timing system and the realization of a mobile station aimed at calibrating the telescopes’ GPS receivers. Studies for the definition of a more suitable and performing GNSS-based timing system would integrate a possible future EEE modernization program and the installation at INRIM of at least two enhanced EEE telescopes accurately oriented to North and with a modular timing system, synchronized to UTC(IT). Finally, the possibility to synchronize to UTC(IT) by means of the White Rabbit protocol (The White Rabbit Official CERN website: https://white-rabbit.web.cern.ch/) over the Italian Quantum Backbone (Insero 2017) some of the EEE Italian telescopes would represent the completion of an example of medium-term constructive collaboration between Time Metrology and Fundamental Physics.

## EEE timing measurement facility at INRIM time and radio navigation laboratories

One of the first steps in the INRIM/CREF collaboration was the realization of a dedicated measurement facility called E3F (i.e., EEE Facility) at INRIM Time Laboratory (Bertacco 2020). It is composed of three low noise time signal distributors (i.e., 1PPS distributors, namely TimeTech PDU and SDI PD5-RM-B), one 40 ps resolution Time Interval Counter (SR620, hereafter indicated as TIC), two dual-frequency GNSS geodetic receivers for timing applications (Septentrio PolaRx5-TR, aka GR03, and Septentrio PolaRx4-TR, aka INR6) and one GPS timing receiver (MESIT GTR50, aka GTRB). Furthermore, the measurement facility is equipped with a dedicated Raspberry Pi 3 Model B + . This minicomputer is used to control and set up all the instruments of the E3F facility (namely the receivers and the TIC) by means of their proprietary software through dedicated connections. Moreover, the Raspberry is in charge of all the data acquisition processes, controlling the measurement execution, and storing the acquired data in the form of text files provisionally. In the case of storage memory consumption, it is connected via Wi-Fi to the INRIM internal network so that data can be copied to different locations.

The scope of this system is to evaluate the instability/accuracy of the 1PPS signals generated by a set of two representative commercial GPS single-frequency receivers installed at EEE telescopes, reproducing GPS Time Scale (GPST, the GPS’s prediction of UTC(USNO) time scale). The receivers are the Spectracom TSync and the Trimble SMT360. In addition, the measurement setup allows for the metrological characterization of the 1PPS signals reproducing GPST, generated by GR03/INR6 receivers, as well as their time-stamp capabilities. Indeed, the goal is to use dual-frequency receivers instead of single-frequency ones in the calibrating traveling station. In the following, TSync, SMT360, GR03 and INR6 will be referred to as DuTs (Devices under Test). For the evaluation of their instability, the 1PPS signals generated by the DuTs are compared with respect to the UTC(IT) 1PPS signal by means of the TIC. As best achievable performance and term of reference, the UTC(IT)-GPST All-in-View (AV) ionosphere-free time difference is computed, by processing with Royal Observatory of Belgium (ROB) R2CGGTTS algorithm (Defraigne 2015) the pseudorange and carrier phase measurements generated by a further calibrated INRIM GNSS dual-frequency receiver (Septentrio PolaRx4-TR, aka INR5). This receiver is regularly used for INRIM time transfer needs. DUTs 1PPS signals accuracy, instead, is mainly evaluated by means of their comparison with respect to GPST, as accessed by the timing receiver GTRB.

While three 1PPS distributors are used to distribute the DuTs signals and have been installed in E3F, only one or two are used at the same time for each measurement campaign. The idea behind this setup is to equip each device involved in the measurement with its own distributor in order to be able to debug the system easily. In addition, the DuTs signals are sent both to the TIC and the GTRB receiver to have simultaneous measurements from two independent systems. Instead, the UTC(IT) 1PPS signal used as a reference is usually sent directly to the TIC. A dedicated distributor is used for this signal only when some calibration or measurement check is required for the whole system. Relative to the TIC, channel A is connected to the reference signal, while channel B to the signal from the DuTs to be measured. The reference signal and the measurand could change depending on the specific measurement campaign. TIC channel B signals are also sent to the GTRB GPS receiver by means of another output of the same distributor. As mentioned above, all the measurement setup is controlled by a Python program developed on a Raspberry Pi 3 Model B + , chosen for its small dimensions, low consumption, and a free operating system. All these characteristics would be useful in a traveling calibration station. The Raspberry Pi is connected to the TIC by an RS232 serial bus and to the GNSS geodetic receiver (either GR03, INR6 or a similar device, depending on the measurement setup) through a USB connection. The Python program acquires the measurement coming from the TIC each second. If a GNSS geodetic receiver is available, each TIC measurement is time-tagged by using the date and time information coming from the NMEA string. If no GNSS is connected to the Raspberry Pi, the time tag is obtained by using the internal NTP protocol of the microcomputer, synchronized with the INRIM Time Laboratory NTP servers. The data acquisition program is being updated in order to take into account possible communication delays and timeouts. However, for the moment, no evidence of similar issues arose. RG58 cables have been used for short interconnections and, depending on the availability at the Laboratory, RG316 ones also. For longer connections, RG128 cables have been preferred because they present lower losses on longer distances.

**Fig. 1:**
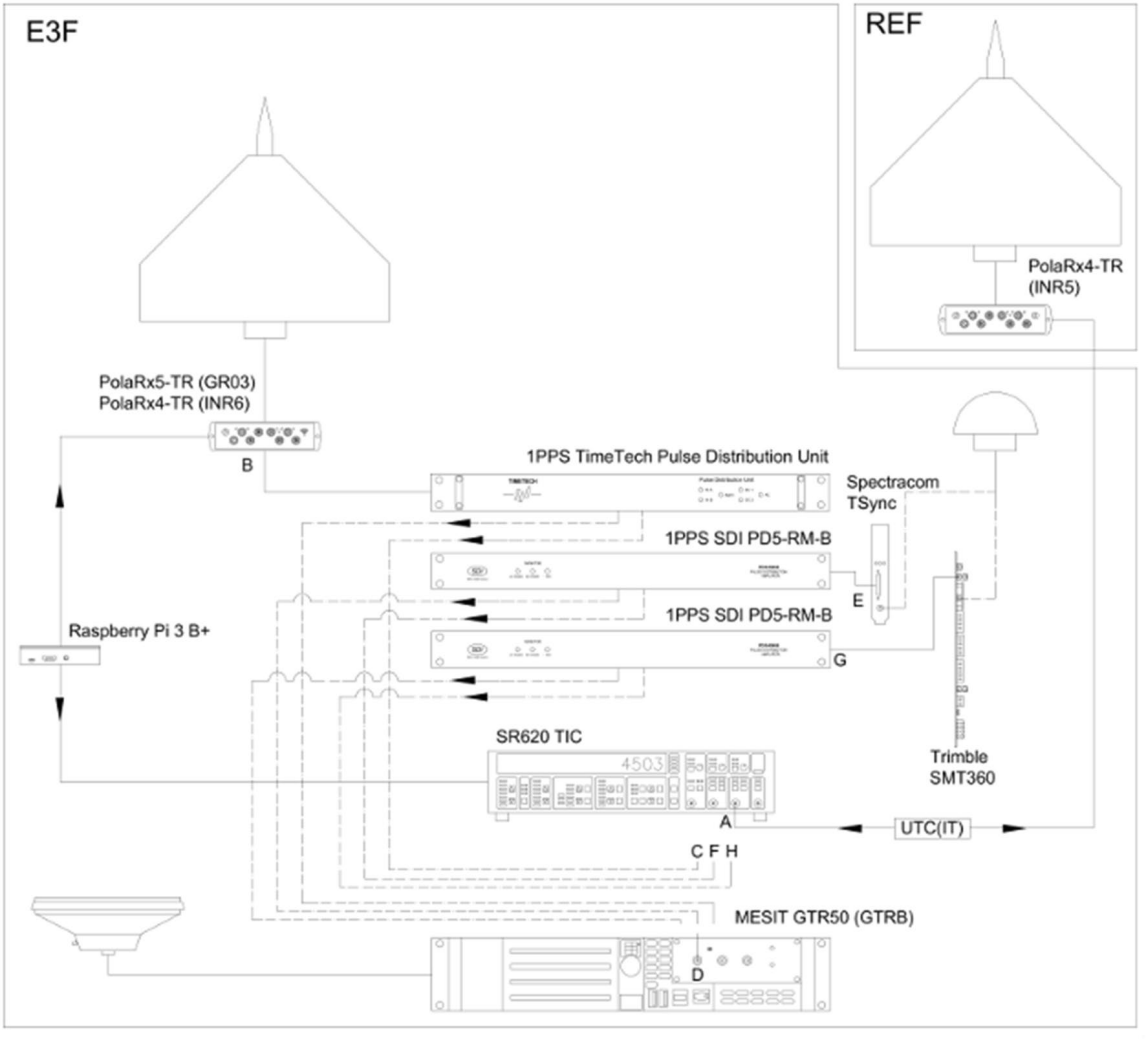
E3F Functional scheme. It is composed by the dual-frequency GNSS receivers PolaRx5-TR (GR03), PolaRx4-TR (INR6 and INR5), and Mesit GTR50 (GTRB); the EEE GPS single-frequency receivers Spectracom TSync and Trimble SMT360; the Time and Frequency distributors 1PPS Time Tech Pulse Distribution Unit and 1PPS SDI PD5-RM-B; the Time Interval Counter (TIC) SR620 and the computer system Raspberry Pi 3 B +

**Fig. 2:**
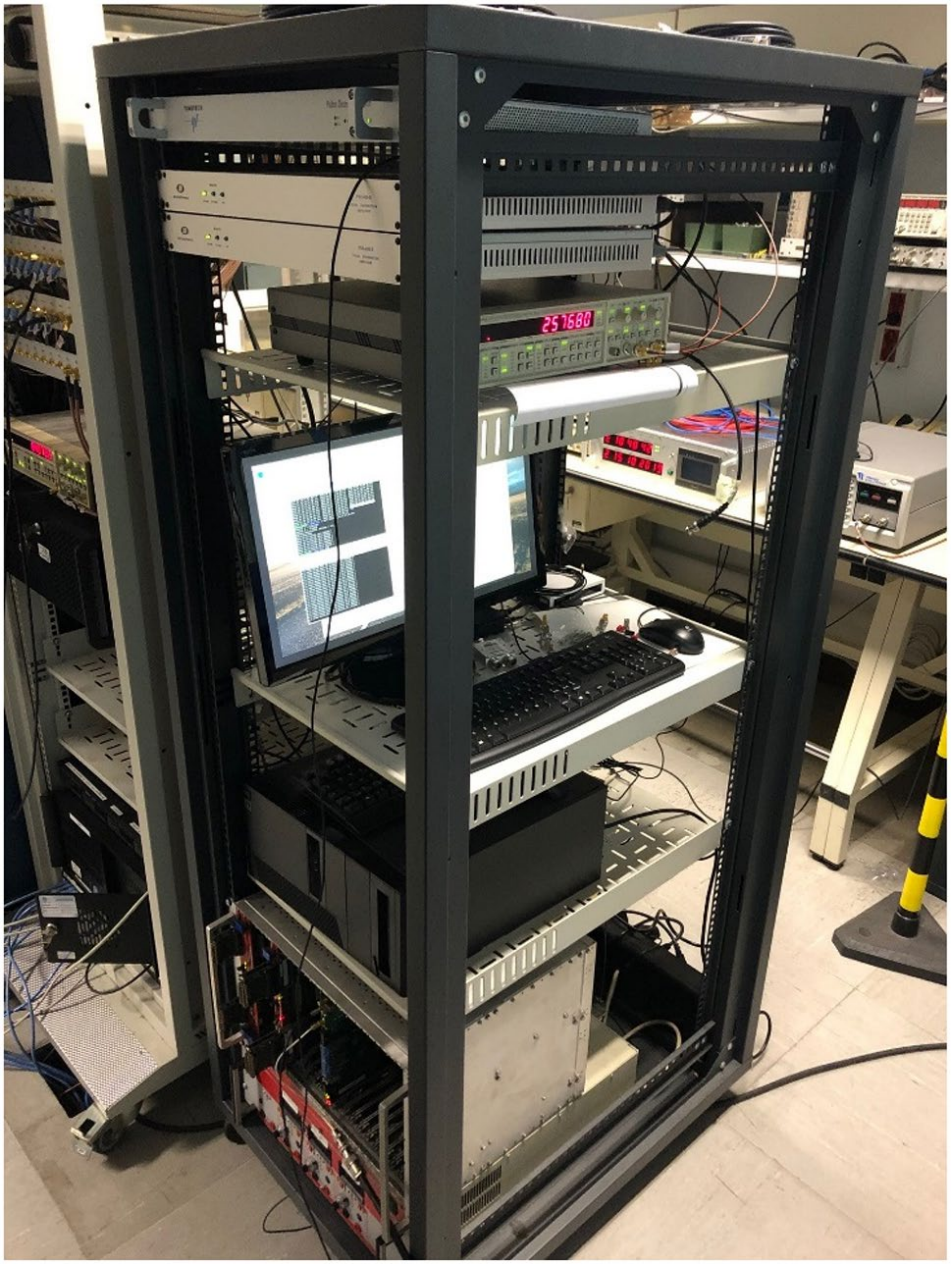
E3F installed, calibrated and operated at INRIM Time Laboratory. From top to bottom, the distribution section, the acquisition section (with the SR620 TIC and, not visible, the Raspberry Pi 3 B + and GR03) and an EEE telescope crate containing the Spectracom TSync and Trimble SMT 360 single-frequency receivers. GTRB and INR5 belong to the Time and Radio Navigation Laboratories measurement systems

**Fig. 3:**
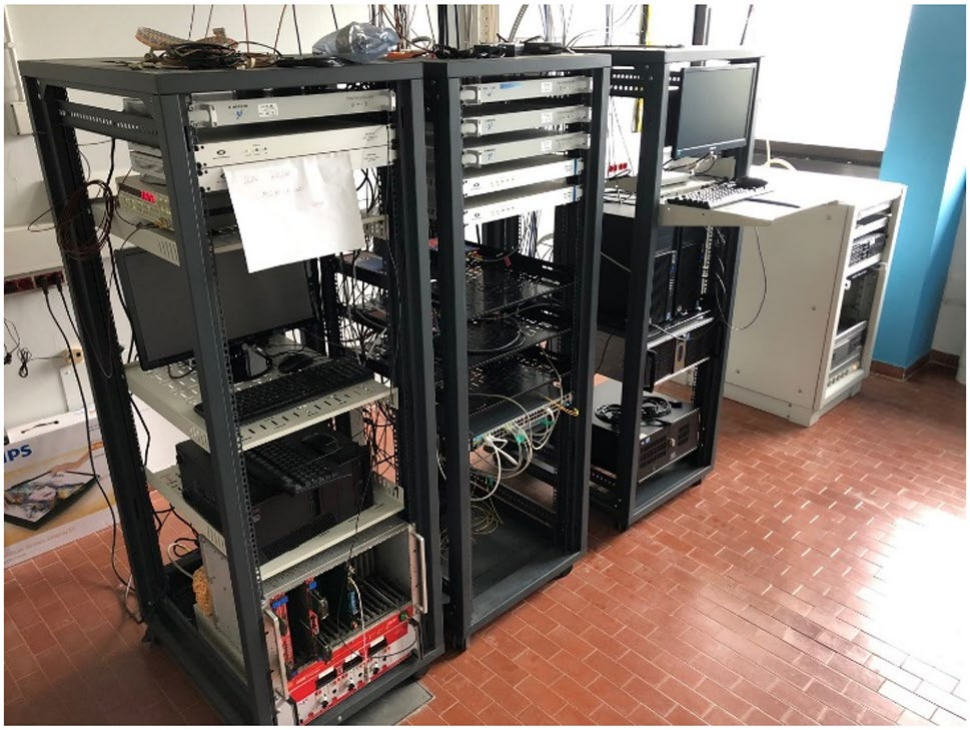
E3F installed, calibrated and operated at INRIM Radio Navigation Laboratory. E3F is the first left-side rack. From top to bottom, the distribution section, the acquisition section (with the SR620 TIC and, not visible, the Raspberry Pi 3 B + and GR03) and an EEE telescope crate containing the Spectracom TSync and Trimble SMT 360 single-frequency receivers. GTRB belongs to the Time Laboratory measurement system, while INR5 is installed in the picture central rack

After its installation at INRIM Time Laboratory, E3F has been fully calibrated, both in terms of distributing devices (i.e., cables and distributors, together with the TIC) and in terms of GNSS receivers (GR03/INR5/INR6 from a 2019 Group2 BIPM differential calibration exercise (Esteban 2015), GTRB internally calibrated). In July 2020, in order to reduce the presence of people at the INRIM Time Laboratory during the Covid-19 emergency, E3F was moved 70 m to the Radio Navigation Laboratory. This operation required the recalibration of UTC(IT) signals. Figure 1 shows the E3F functional scheme, while Figs. 2 and 3 show E3F installation at both the INRIM Time and Radio Navigation Laboratories.

## Preliminary measurement results

The first E3F application is related to the instability/accuracy evaluation of the 1PPS signals generated by TSync and SMT360, the two EEE representative GPS single-frequency receivers. These 1PPSs reproduce GPST and represent the time reference for the triggering system of the EEE telescopes. The same measurement exercise is then repeated for GR03 and INR6, in order to evaluate their time-stamp capabilities.

GR03 is currently at the top of the range among INRIM receivers and is nominally able to reproduce a 1PPS signal synchronized with GPST, with nominally good characteristics in terms of instability. This 1PPS is also accurate if the System Delay (SYS DLY, composed by the antenna, antenna cable and receiver delays) is estimated and applied at the receiver level or in post processing.

INR6 is an earlier model than GR03, and its internal system for the generation of a 1PPS reproducing GPST depends on how it is operated. If the receiver is operated in a free-running mode, the internal clock is frequency locked to GPST through the time information of the PVT solution, but it is not synchronized with the system time scale. This means that 1PPS output is the receiver internal clock 1PPS, closest to the real GPST (the estimated one), while the residual is recorded into a receiver memory field called xPPSoffset, which can be retrieved from the Septentrio Binary Files (i.e., SBF) and post-processed. Suppose the receiver is operated connected to an external time/frequency reference by means of 1PPS and 10 MHz (e.g., UTC(k)). In that case, the output is still the internal clock 1PPS closest to the real GPST, but stable as the external reference, and the residual is still recorded into the xPPSoffset. Concerning the SYS DLY applied at the receiver level, it is important to mention that the physical 1PPS output is offset only by multiple integer cycles of the internal clock frequency. For INR6 it is 20 ns, being the internal clock frequency 50 MHz, while for GR03 it is 33 ns (the clock frequency is 33 MHz). Finally, the fractional part is included in the xPPSoffset. Starting from these assumptions, the E3F measurements scheme reported in Table 1 was conceived in order to perform the preliminary evaluation of the current EEE timing system.

**Table 1:** E3F measurement setups for the preliminary evaluation of the EEE timing system

Setup	GNSS	Mode of Operation	Calibration	SYS DLY use	Measurement Period
1	TSync	Free-running	Uncalibrated	NA	MJD 58,763.5–58,766.5
2	SMT360	Free-running	Uncalibrated	NA	MJD 58,767.3–58,770
3	GR03	Free-running	Calibrated	Receiver	MJD 58,759.4–58,763
4	INR6	Free-running	Calibrated	Receiver	MJD 59,024–59,029
5	INR6	UTC(IT) referenced	Calibrated	Receiver	MJD 59,046–59,050
6	INR6	UTC(IT) referenced	Calibrated	Computation	MJD 59,054–59,057

For the sake of completeness and clarity of presentation, the first part of this section provides results very recently presented (Cerretto 2020) for the measurement Setups #1, #2, and #3, and the second part new results for setups #4, #5 and #6, as well as for the E3F metrological confirmation after its transfer to the INRIM Radio Navigation Laboratory.

Table 2 and Table 3 summarize the results for all the setups, differentiating the UTC(IT)-1PPS(GPST) and 1PPS(GPST)-GPST baselines, as well as the modes of operation and SYS DLY application strategy.

**Table 2:** Measurement results for the UTC(IT)-1PPS(GPST) time difference, receivers modes of operation, and SYS DLY application strategy are reported

Setup	Measurement	Avg (ns)	StDev (ns)	xPPS offset (Avg/StDev ns)
1	UTC(IT)-1PPS(GPST) [TIC]	296.92	12.64 ns	–
	UTC(IT)-1PPS(GPST) [INR5/GTRB AV]	296.29	10.41 ns	–
	UTC(IT)-GPST [INR5 AV]	− 0.29	0.74 ns	–
2	UTC(IT)-1PPS(GPST) [TIC]	241.87	8.92 ns	–
	UTC(IT)-1PPS(GPST) [INR5/GTRB AV]	240.67	6.08 ns	–
	UTC(IT)-GPST [INR5 AV]	0.77	0.64 ns	–
3	UTC(IT)-1PPS(GPST) [TIC]	− 4.46	1.78 ns	–
	UTC(IT)-1PPS(GPST) [INR5/GTRB AV]	− 4.67	1.62 ns	–
	UTC(IT)-GPST [INR5 AV]	− 4.99	0.83 ns	–
4	UTC(IT)-1PPS(GPST) [TIC]	11.59 ns	2.04 ns	–
	UTC(IT)-1PPS(GPST) + xPPS [TIC]	− 0.35 ns	1.73 ns	6.20 ns/1.40 ns
	UTC(IT)-GPST [INR5 AV]	2.33 ns	1.40 ns	–
5	UTC(IT)-1PPS(GPST) [TIC]	3.73 ns	0.80 ns	–
	UTC(IT)-1PPS(GPST) + xPPS [TIC]	− 2.61 ns	1.45 ns	0.57 ns/1.44 ns
	UTC(IT)-GPST [INR5 AV]	0.34 ns	0.95 ns	–
6	UTC(IT)-1PPS(GPST) [TIC]	− 2.03 ns	0.08 ns	–
	UTC(IT)-1PPS(GPST) + xPPS + SYS DLY [TIC]	− 0.64 ns	2.18 ns	− 1.49 ns/2.18 ns
	UTC(IT)-GPST [INR5 AV]	− 3.08 ns	1.26 ns	–

UTC(IT)-GPST via INR5 AV to be considered as term of reference, while average values for Setup #3 reflect a 1.33 ns/day rate in the UTC(IT) time scale for the considered period

**Table 3:** Measurement results for the 1PPS(GPST)-GPST time difference. Receiver’s modes of operation and SYS DLY application strategy are reported

Setup	Measurement	Average (ns)	StDev (ns)	xPPS offset (Avg/StDev ns)
1	1PPS(GPST)-GPST [GTRB AV]	296.38 ns	10.30 ns	–
2	1PPS(GPST)-GPST [GTRB AV]	239.95 ns	6.12 ns	–
3	1PPS(GPST)-GPST [GTRB AV]	0.52 ns	1.52 ns	–
4	1PPS(GPST)-GPST [GTRB AV]	− 10.30 ns	1.54 ns	–
	1PPS(GPST)-GPST + xPPS [GTRB AV]	1.69 ns	2.10 ns	6.20 ns/1.10 ns
5	1PPS(GPST)-GPST [GTRB AV]	− 4.70 ns	0.95 ns	–
	1PPS(GPST)-GPST + xPPS [GTRB AV]	1.46 ns	1.58 ns	0.20 ns/1.46 ns
6	1PPS(GPST)-GPST [GTRB AV]	2.20 ns	1.17 ns	–
	1PPS(GPST)-GPST + xPPS + SYS DLY [GTRB AV]	− 3.65 ns	2.52 ns	− 1.45 ns/2.23 ns

Setup #1 and Setup #2 show the 1PPS signal reproducing GPST and generated by both the TSync and SMT360 receivers, compared to UTC(IT) by means of the TIC and the INR5/GTRB AV combination. The comparison of the receivers 1PPSs with respect to GPST, as accessed by GTRB, is also shown. This measurement exercise yield to estimate an instability for the TSync and SMT360 receivers 1PPS output of 12.64 ns (1σ) and 8.92 ns (1σ), respectively, together with a no time consistence with zero in the 1PPS-GPST time difference (an offset of 296.38 ns and 239.95 ns, respectively, can be depicted). This result confirms that GPS receivers deployed at the EEE network are not particularly stable and—above all—not calibrated. For SMT360, pronounced typical periodicities due to the ionosphere effect are also present.

After having tested the time-stamping performances of the two representative EEE single-frequency receivers, the 1PPS signal reproducing GPST and generated by GR03, is compared with respect to UTC(IT) by means of the TIC and the INR5/GTRB AV combination, as well as with respect to GPST, as accessed by GTRB. Setup #3 shows the good stability of the GR03 1PPS (1.78 ns (1σ)), together with the time difference with respect to GPST consistency with zero (residual of 0.52 ns). This means that GR03 is able to generate a 1PPS signal reproducing GPST, with notable characteristics in terms of stability and accuracy.

Setup #4 to Setup #6 show the results of the time-stamp capabilities of INR6, considering both modes of operation and applying SYS DLY at receiver level and in post processing. This last distinction is taken into account to verify the receiver’s capability to shift the 1PPS output, depending on how the SYS DLY value is applied.

At first, in Setup #4, the instability evaluation of INR6 1PPS output reproducing GPST is performed considering the UTC(IT)-GPST baseline, when the receiver is operated in free-running mode and SYS DLY applied at receiver level. These measurements are obtained by means of the E3F TIC (channel A connected to UTC(IT) and channel B connected to the INR6 1PPS output signal) and are compared with the equivalent quantity obtained by processing the INR5 code/phase measurements with the ORB R2CGGTTS algorithm. The measurement contained in the xPPSoffset field is used in combination with the TIC ones, yielding to an instability that can be stated at a level of 1.73 ns (1σ), comparable with the ones related to GR03 (1.78 ns (1σ)). Then, the time-stamp capabilities are evaluated comparing receiver 1PPS output with respect to GPST, as assessed by means of GTRB. The measurement contained in the xPPSoffset field is used in combination with the GTRB measurements. Here, a good agreement is shown, although a 1.69 ns time offset is still present. This offset comes from a residual in the GTRB measurements (in the order of 1.13 ns), as it will be shown at the end of this section.

The same exercise results, but with INR6 referenced to UTC(IT) time scale (through its 1PPS and 10 MHz signals) and with SYS DLY applied at receiver level, are then presented. For Setup #5, the computational procedure is the same as Setup #4, with the xPPSoffset field measurements applied to the TIC and GTRB estimates. Here, the instability of the INR6 1PPS output signal reproducing GPST can be appraised at a level of 1.45 ns (1σ), still comparable with the one of GR03 (1.78 ns (1σ)), while the agreement with GPST as assessed with GTRB, still shows the GTRB misalignment mentioned above (residual of 1.46 ns).

Finally, the results of the same exercise, but with INR6 still referenced to UTC(IT) time scale and SYS DLY applied at the software level, are presented. Also, in Setup #6, the computational procedure is the same as the previous setups, with the xPPSoffset field measurements applied to the TIC and GTRB estimates, together with the SYS DLY value. Here, the instability of the INR6 1PPS signal reproducing GPST, can be appraised at a level of 2.18 ns (1σ), still comparable with the ones related to GR03 (1.78 ns (1σ)), while the agreement with GPST as assessed with GTRB, seems to be slightly bigger than in the previous configurations (residual of − 3.65 ns).

Furthermore, an acceptable time alignment between E3F measurements and the INR5 ones (considered as reference) is still evident, but with a time offset ranging from 2.44 ns to 2.95 ns, depending on the configuration.

**Fig. 4:**
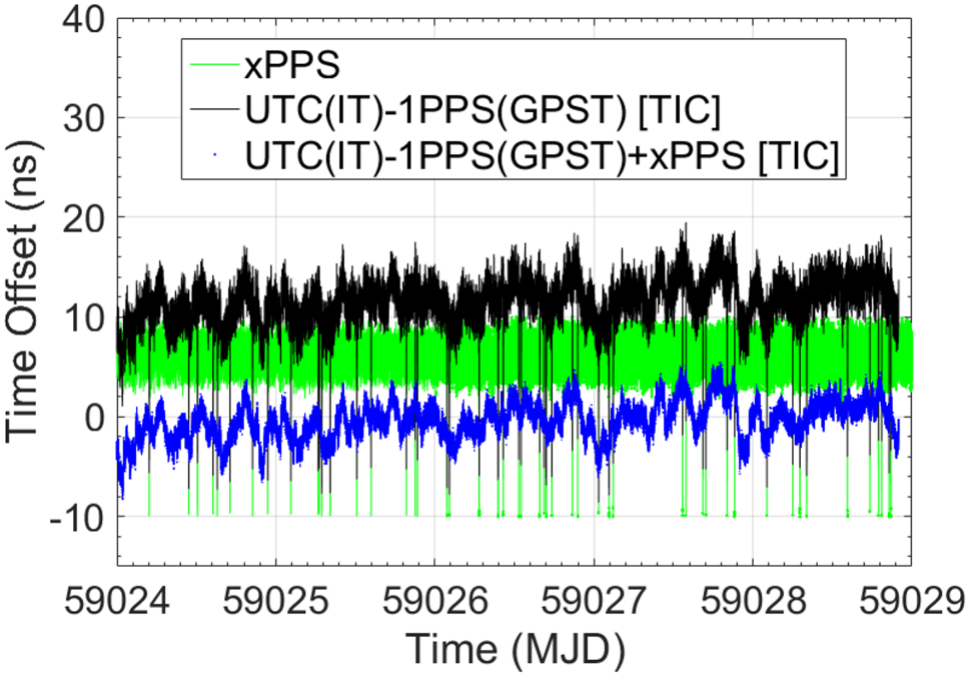
INR6 in free-running mode of operation. Complementary 20 ns cycle slips affecting TIC and xPPS measurements are depictable. From the combination of both measurements, cycle slips are removed

**Fig. 5:**
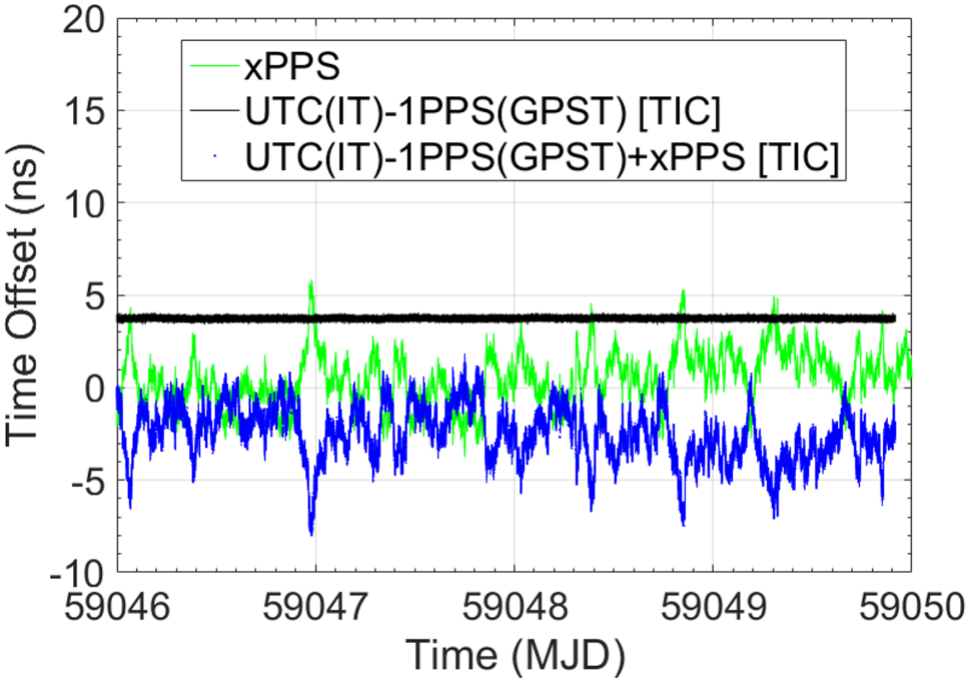
INR6 in UTC(IT) referenced mode of operation and SYS DLY applied at receiver level. Improved stability in the TIC measurements and absence of cycle slips are depictable

**Fig. 6:**
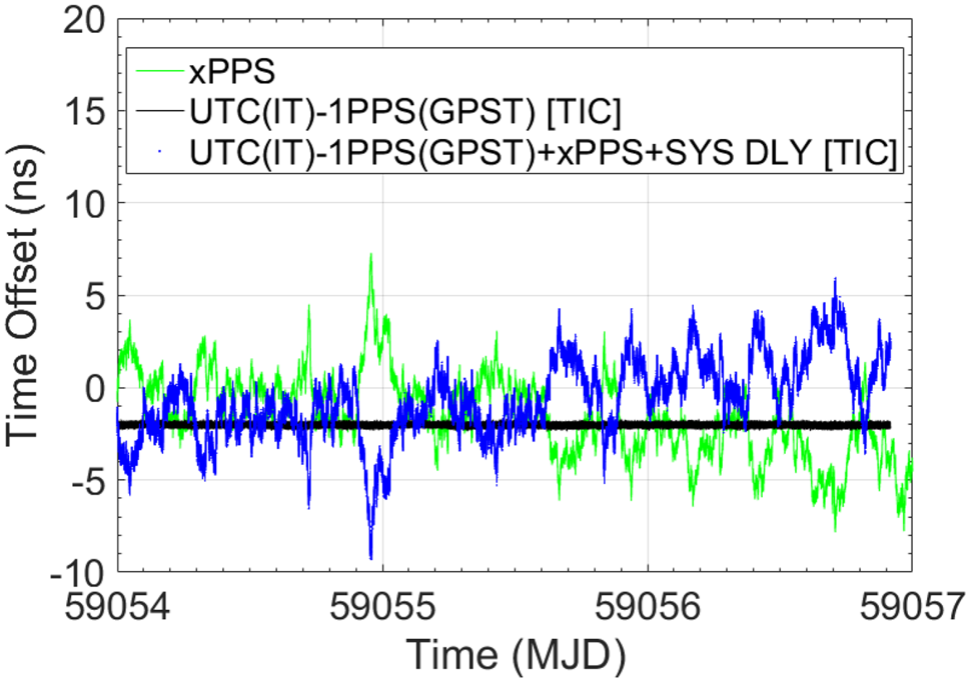
INR6 in UTC(IT) referenced mode of operation and SYS DLY applied at the software level. Improved stability in the TIC measurements and absence of cycle slips are depictable

In parallel, another aspect to be highlighted, is the behavior of the xPPSoffset measurements and the TIC measurements instabilities. In Fig. 4, 20 ns spikes can be depicted in the TIC measurements, as well as complementary ones in the xPPSoffset. This behavior comes from the fact that the 1PPS output is the internal clock 1PPS, closest to the real GPST. Depending on the closeness, 20 ns cycle slips could occur and be measured by the TIC (receivers internal clock frequency is 50 MHz). Of course, complementary spikes are induced in the xPPSoffset and removed by combining both measurements. In Figs. 5 and 6, the TIC and xPPSoffset measurements do not show the 20 ns spikes anymore because the internal clock of the receiver is synchronized and frequency locked by the UTC(IT) 1PPS and 10 MHZ reference signals, respectively. In fact, also here, the 1PPS output is the internal clock 1PPS closest to the real GPST, but in this case, tracing for the stability of the external reference and not suffering for the 20 ns cycle slips. Please, note the improved TIC instability, with respect to the previous case, when INR6 is operated in free-running mode.

**Fig. 7:**
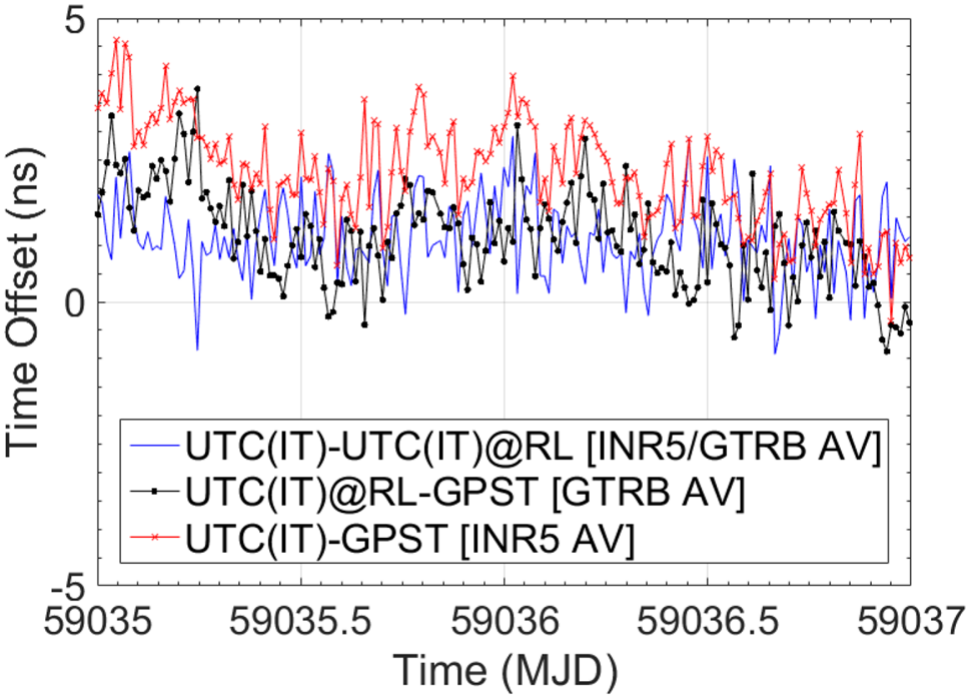
E3F metrological confirmation after its move from the Time Laboratory to the Radio Navigation Laboratory (RL). The calibration evaluation for the E3F GPS component is reported

**Fig. 8:**
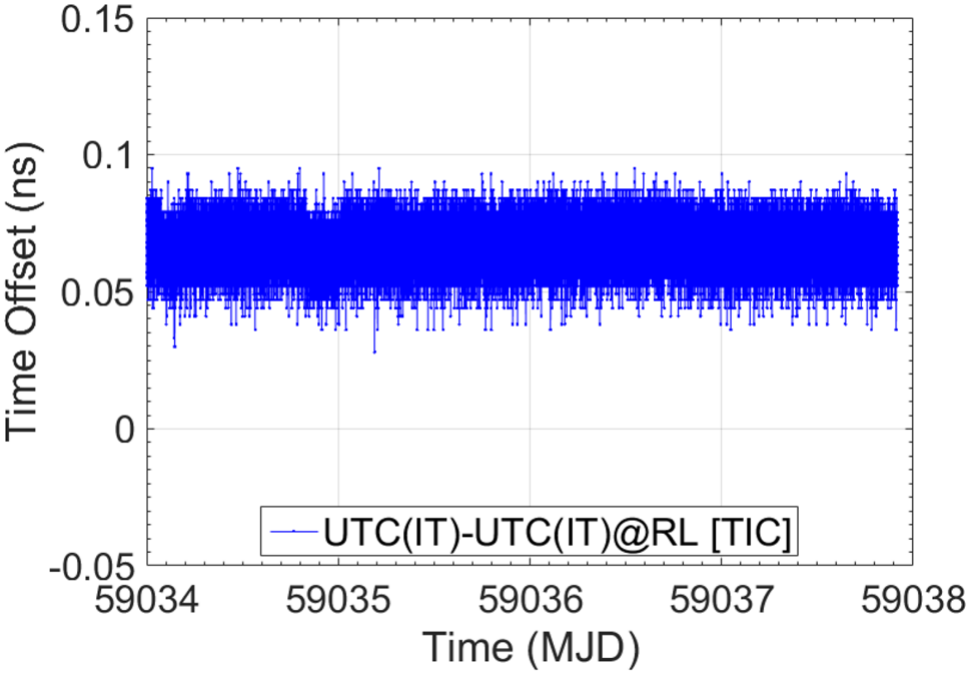
E3F metrological confirmation after its move from the Time Laboratory to the Radio Navigation Laboratory (RL). The calibration evaluation for the E3F TIC, distributors and cables is reported

Finally, in Figs. 7 and 8, a metrological confirmation of E3F after its transfer from the INRIM Time Laboratory to the Radio Navigation one is reported. The confirmation, which has been performed considering UTC(IT) at Time Laboratory as a reference, together with UTC(IT) as distributed at INRIM Radio Navigation Laboratory as the measurand, shows a misalignment of 1.13 ns in the INR5/GTRB AV measurements (issue still under investigation) and a complete alignment in the TIC ones. Table 4 resumes the E3F metrological confirmation results after its move from the Time Laboratory to the Radio Navigation Laboratory.

**Table 4:** Results of E3F metrological confirmation after its move from the Time Laboratory to the Radio Navigation Laboratory (RL)

Measurement	Measurement Period	Average (ns)	StDev (ns)
UTC(IT)-UTC(IT)@RL [INR5/GTRB AV]	MJD 59,035–59,037	1.13	0.73
UTC(IT)-UTC(IT)@RL [TIC]	MJD 59,034–59,038	0.07	0.06

## Traveling calibrating station for EEE telescopes: a preliminary strategy

In the previous section, the characteristics of TSync and SMT360 were shown, together with the time-stamp capabilities of two GNSS geodetic receivers for timing applications (i.e., GR03 and INR6). The latter result suggests that using the GR03 receiver, generating a calibrated 1PPS signal reproducing GPST, can be considered an accurate and stable reference for the EEE GPS receiver’s 1PPS signals. In addition, the use of INR6 can be taken into account, but here the management of the xPPSoffset is required. Independently from the receiver, the station would be equipped with a dedicated computer system. Alternatively, a self-consistent GPS receiver for timing applications, like GTRB, could be considered a compact unit devoted to the calibration of the EEE GPS stations.

## INRIM laboratories dedicated to EEE and possible links to the Italian Quantum Backbone map

For the next future is foreseen to host INRIM at least two enhanced EEE telescopes to be considered as a reference for the EEE network. In such a direction, the preparation of two dedicated INRIM Laboratories has been envisaged. The space for the first laboratory has already been selected and, compatibly with Covid-19 emergency rules, some preliminary work started. Each laboratory must be located on high floors, reclaimed from Radio Frequency (RF) noise and with a good power supply grounding system up to 100 MHz, in order to avoid possible reflections. Furthermore, the laboratory needs to be provided with a stable temperature (e.g., 20 ± 1 °C), a customized safe gas distribution system and a floor withstanding 350 kg over 4 × 20 cm^2^ corners. The gas distribution installation has begun, taking benefit of a brand-new INRIM general distribution system under realization for the same building where the first INRIM EEE Laboratory will be located. This system will allow the distribution of the gas currently employed for the EEE telescopes and the possibility of using a second generation of gas mixture, which is cheaper and quality efficient, based on Carbon Dioxide and Argon. Having the environment and telescopes gas mixture Pressure (P), Temperature (T), and Humidity (H) to be monitored, a calibrated and precise measurement system needs to be implemented, possibly benefiting from the previous experience at INRIM in this field. A modular timing system directly synchronized with UTC(IT) by means of coaxial cables and optical fibers, together with an accurate orientation to North, will be one of the breaking aspects aimed at considering INRIM telescopes as a reference, among the ones belonging to the EEE network. Furthermore, the Laboratories are designed in order to be potentially considered support facilities, allowing timing studies for Particle Physics/Astrophysics experiments, besides EEE. Finally, it will also be envisaged the possibility to synchronize some of EEE Italian telescopes directly to UTC(IT), by means of the Italian Quantum Backbone, over White Rabbit Protocol.

## Conclusions

A formal collaboration has been set up between INRIM and CREF to perform the metrological characterization and optimization of the EEE timing system. It was found that metrological-level characterization is extremely vital for proper astronomical functioning, and that the standard GPS receivers installed in EEE telescopes were not adequate in terms of time accuracy and stability. Instead, carefully calibrated dual-frequency GNSS receivers are required for the EEE experiment to reach its full potential. Through use of metrological techniques, including carefully periodic calibration campaigns, it is possible to achieve a reduction of 100’s of nanoseconds in systematic offsets and of an order of magnitude in the statistical errors. Although slowed by the Covid-19 pandemic, progress has been made in designing a fully equipped support laboratory and a portable calibration system. The initial efforts described herein are to be viewed as merely the beginning of a long-term collaboration that will provide useful astronomical data while simultaneously introducing and engaging students and elementary-particle physicists to metrological science.

## Data Availability

The datasets generated during and/or analyzed during the current study are available from the corresponding author on reasonable request.
